# The Propensity of Pentatricopeptide Repeat Genes to Evolve into Restorers of Cytoplasmic Male Sterility

**DOI:** 10.3389/fpls.2016.01816

**Published:** 2016-12-06

**Authors:** Lydiane Gaborieau, Gregory G. Brown, Hakim Mireau

**Affiliations:** ^1^Department of Biology, McGill UniversityMontreal, QC, Canada; ^2^Institut Jean-Pierre Bourgin, INRA, AgroParisTech, CNRS, Université Paris-SaclayVersailles, France

**Keywords:** fertility restorer, CMS, mitochondria, PPR proteins, RNA

## Abstract

Cytoplasmic male sterility (CMS) is a widespread phenotype in plants, which present a defect in the production of functional pollen. The male sterilizing factors usually consist of unusual genes or open reading frames encoded by the mitochondrial genome. CMS can be suppressed by specific nuclear genes called restorers of fertility (*Rf*s). In the majority of cases, *Rf* genes produce proteins that act directly on the CMS conferring mitochondrial transcripts by binding them specifically and promoting processing events. In this review, we explore the wide array of mechanisms guiding fertility restoration. PPR proteins represent the most frequent protein class among identified Rfs and they exhibit ideal characteristics to evolve into restorer of fertility when the mechanism of restoration implies a post-transcriptional action. Here, we review the literature that highlights those characteristics and help explain why PPR proteins are ideal for the roles they play as restorers of fertility.

## Introduction

Cytoplasmic male sterility (CMS) has been characterized in over 140 natural species (Laser and Lersten, 1972). In nature, CMS can be observed in gynodioecious natural populations, where hermaphrodite and female (male sterile) plants coexist within a same group (Charlesworth, 2002; Touzet and Budar, 2004). The male sterilizing factors are produced by recombination of the mitochondrial genome and consist of unusual genes or open reading frames (ORFs) that usually contain a portion of functional mitochondrial genes and sequences of unknown origin (Hanson and Bentolila, 2004). These unusual ORFs are maternally inherited, and effectively translated into novel mitochondrial proteins, with the resulting failure to produce functional pollen as the sole observed phenotype (Chen and Liu, 2014). Theoretical models have indicated that CMS ORFs may be maintained in natural populations by their ability to make female plants reproductively more successful [more seeds are produced by male-sterile plants than hermaphroditic individuals (Budar et al., 2003)]. CMS can be suppressed by specific nuclear genes called restorers of fertility (*Rf*), which restore partial to normal pollen production to plants carrying a corresponding CMS-inducing cytoplasm. The importance of studying such systems in the last few decades resides in how they deepen our understanding of the interactions between the nuclear and mitochondrial genomes as well as the importance CMS has in applied agriculture (e.g., in hybrid production).

In the majority of cases, *Rf* genes produce proteins that bind specifically to the CMS conferring transcripts in the mitochondria and promote processing events leading to a strong reduction in the production of mitochondrial CMS-inducing proteins (reviewed in Chen and Liu, 2014). In recent years, a majority of the proteins encoded by *Rf* genes have been found to belong to the PPR (Pentatricopeptide Repeat) family (Aubourg et al., 2000; Small and Peeters, 2000; Dahan and Mireau, 2013). This protein family is largely expanded in land plant genomes. PPR proteins have in common a canonical P-type 35 amino acid domain repeated in tandem up to 30 times. Length variations of that original P-type PPR domain allowed the creation of longer (L-type) or shorter (S-type) domains for which some variants were recently identified (Lurin et al., 2004; Cheng et al., 2016). The PPR protein family is consequently divided in subfamilies depending on the number and type of repeats present in their sequence as well as optional C-terminal domains. P-type PPR proteins participate in various aspects of organellar RNA processing ranging from transcription to translation, whereas C-to-U RNA editing appeared to be the major function of PLS PPR proteins (for review see Barkan and Small, 2014; Hammani and Giegeè, 2014).

Recent three-dimensional structural analyses of PPR proteins confirmed that each repeat is configured as two anti-parallel helices (Ban et al., 2013; Gully et al., 2015). Because the repeats are repeated several times within a protein, the succession of PPR domains gives a general rectangular form to the protein with one side highly positively charged that is involved in RNA binding (Ban et al., 2013; Ke et al., 2013; Yin et al., 2013), confirming early hypotheses on PPR protein mode of action (Bentolila et al., 2002; Kotera et al., 2005; Schmitz-Linneweber et al., 2005). Gel mobility shift assays showed that the P-type PPR domain has a higher affinity for single-stranded RNA compared to single and double-stranded DNA molecules supporting the RNA binding capacity of these proteins (Williams-Carrier et al., 2008).

Within one PPR domain some amino acids are of greater importance for RNA recognition. Several studies have demonstrated the existence of a recognition code between the identity of specific amino acids within the repeats and the target RNA sequence of the PPR protein studied (Barkan et al., 2012; Takenaka et al., 2013; Yagi et al., 2013); the identity of the 5th and the 35th amino acids of each motif have been shown to be particularly important (residue numbering according to Yin et al., 2013 and discussed in Cheng et al., 2016). These two amino acids are generally positively charged (Small and Peeters, 2000; Ban et al., 2013). The binding between these amino acids and the target nucleotide has been experimentally proven in the case of the PPR10 (Barkan et al., 2012), the THA8 (Ke et al., 2013), the CLB19 (Kindgren et al., 2015; Ramos-Vega et al., 2015) and the AEF1/MPR25 (Yap et al., 2015) PPR proteins. In the context of CMS, where Rf proteins process unusual transcripts, nucleic acid specificity is essential to specifically target the CMS-conferring transcript.

Recent studies revealed that the mechanisms by which PPR proteins recognize their target RNAs are highly specific but also revealed a certain level of flexibility (Barkan et al., 2012; Yagi et al., 2014). That flexibility allows PPR proteins to bind multiple RNAs. Additionally, some *PPR* genes are governed by evolutionary factors that facilitate their diversification and duplication in a relatively short time scale. In this review, we will highlight some of these peculiarities and discuss how *PPR* genes make ideal candidates to rapidly evolve as fertility restorer genes. Consequently, the main topic of this review is about *Rf* genes and mitochondrial CMS-causing genes are only briefly introduced in the text. We encourage readers who want more details on CMS genes to refer to specialized reviews (Hanson and Bentolila, 2004; Chen and Liu, 2014).

## Restorer Proteins Belonging to the PPR Family

The first identified *Rf* gene acting on a CMS transcript was *Rf-PPR592* from Petunia (**Table 1**, Bentolila et al., 2002). Petunia CMS is caused by the expression of the *pcf* (petunia CMS fused) mitochondrial ORF. The *pcf* gene is composed of portions of two standard mitochondrial genes, *atp9* and *cox2*, as well as a sequence of unknown origin (Young and Hanson, 1987). The petunia restorer locus contains two genes *PPR591* and *PPR592*, of which only PPR592 carries restoration activity (Bentolila et al., 2002). When the sequences within and around these two *PPR* genes, which are present in both restoring and non-restoring genotypes, were compared, no changes in the coding sequences of the genes were found, but a mutation in the promoter of PPR592 in the CMS genomic background prevented its expression in floral buds (Bentolila et al., 2002). PPR592 was shown to rescue fertility by altering the *pcf* transcript profile and dramatically reducing the quantity of PCF protein present in the mitochondria (Bentolila et al., 2002). Immunoprecipitation experiments of mitochondrial fractions (Gillman et al., 2007) demonstrated that PPR592 is associated with the inner membrane of the mitochondria in a large protein complex that binds *pcf* RNA, indicating that a number of partner proteins in addition to PPR592 are involved in the restoration process.

**Table 1:** Representation of the characterized restorers of fertility (confirmed through transgenic analysis) and their updated model for restoration mechanism.

	Restorer name	Type of restorer protein	Species	CMS name	CMS inducing gene	Characteristics and models for fertility restoration	Reference
Restoration by post-transcriptionnal events	Rf-PPR592	PPR protein	Petunia	–	*pcf*	Binds to CMS transcript Alters transcript expression profile In a large complex with CMS transcript associated with Inner mitochondrial membrane	Bentolila et al., 2002; Gillman et al., 2007
	
	Rfo	PPR protein	Radish	Ogura	*orfl38-orfB*	Binds to CMS transcript Does not promote processing or degradation Reduced levels of CMS protein in restored plants	Brown et al., 2003; Desloire et al., 2003; Koizuka et al., 2003; Uyttewaal et al., 2008; Qin et al., 2014
	
	Rf1A, Rf1B	PPR protein	Rice	BT	*atp6-orf79*	Mediation of endonucleolytic processing of cms transcript Destabilization of dicistronic transcript	Wang et al., 2006
	
	Rf4, Rf3	PPR protein	Rice	Wild Abortive	*WA352*	Reduced levels of CMS conferring transcript	Tang et al., 2014
	
	Rf5, GRP162, Rf6	PPR protein + glycine-rich protein	Rice	Honglian	*atp6-orfH79*	PPR not binding CMS transcript Translation inhibition by GRP162 GRP162 + Rf5 induces transcript processing	Hu et al., 2012, 2013
	
	PPR762	PPR protein	Rice	RT98-type	*orf113*	Found to process *orf113* Only very partially restores fertility	Igarashi et al., 2016
Restoration by other mechanisms	Rf2	Glycine-rich protein	Rice	Lead	*?*	Within a large protein complex Targetting the CMS protein	Itabashi et al., 2011; Fujii et al., 2014
	
	Rf2	ALDH protein	Maize	Texas	*urf13*	Remove oxidative stress caused by URF13	Liu et al., 2001
	
	Rf17	Acyl carrier protein	Rice	CW	?	Retrograde regulation Diminution of expression levels of Rf17 induces metabolic Alteration in mitochondria that leads to restoration	Fujii and Toriyama, 2009
	
	bvORF20	OMA1-like protein	Beet	Owen	*preSatp6*	Interaction with CMS polypeptide Impact the homo-oligomerization of the CMS peptide in mitochondrial membranes	Kitazaki et al., 2015

*To allow better visualization, highlighting of the PPR proteins characterized as restorers acting at the post-transcriptional level was done in contrast to other restorers characterized in other processes.*

Following the characterization of PPR592, a number of other restorers of fertility genes were found to encode PPR proteins. The radish (*Raphanus sativus)* restorer gene for Ogura CMS (Rfo) was cloned through a map-based cloning approach that relied, in part, on the synteny that exists between the sequenced *Arabidopsis thaliana* and the radish genome (Brown et al., 2003; Desloire et al., 2003; Koizuka et al., 2003). In radish, Ogura CMS is associated with the co-transcription of two ORFs *orf138* and *orfB* (*atp8*, see **Table 1**). *orf138* is the sterility-inducing gene and *orfB* encodes the subunit eight of the ATP-synthase complex (Bonhomme et al., 1992; Heazlewood et al., 2003). Within the restoration locus *Rfo*, three predicted genes *PPR-A, PPR-B* and *PPR-C* encode proteins belonging to the PPR family (Desloire et al., 2003). *PPR-C* was later found to be a pseudogene and only *PPR-B*, now confirmed as the *Rfo* gene, restored CMS by down-regulating the expression of ORF138 (Brown et al., 2003; Koizuka et al., 2003). Not only expression levels of *Rfo* were found to be higher than *PPR-A* (Uyttewaal et al., 2008; Qin et al., 2014), but *Rfo* was also found to specifically affect the expression of *orf138* in the tapetum of anthers, suggesting a tissue specific action (Uyttewaal et al., 2008). Further insight into the restoration mechanism was gained by co-immunoprecipitation experiments with *orf138* RNA (Uyttewaal et al., 2008). According to data from that study, Rfo binds specifically to the *orf138* transcript but does not promote the processing or the degradation of the CMS conferring RNA, even at a tissue specific level. The current model about the function of *Rfo* suggests that the restoration occurs by the blockage of the translation of the CMS conferring transcript, *orf138* (Uyttewaal et al., 2008).

BT-rice CMS provides another example in which PPR proteins are involved in the fertility restoration mechanism (**Table 1**). The sterility in BT-rice is associated with the presence of a large transcript composed of *atp6* sequences co-transcribed with a downstream novel ORF, *orf79*, that is composed of sequences derived from *cox1* and a sequence of unknown origin (Akagi et al., 1994). Positional cloning of the fertility restoration locus revealed that it contained nine *PPR* genes (Akagi et al., 2004). Two of those *PPR* genes, *Rf1A* and *Rf1B*, appear to be recently duplicated ORFs and both show fertility restoration capacity for the BT CMS but employ different mechanisms (Wang et al., 2006). Wang et al. (2006) showed, by RNA gel blot experiments, that Rf1A induces a reduction in *orf79* transcript levels, and circular RT-PCR (cRT-PCR) experiments demonstrated that Rf1A governed the appearance of smaller transcripts with 5′ ends produced by RNA cleavage events. Rf1A is therefore thought to act by the mediation of specific endonucleolytic cleavage within *orf79*. On the other hand, in the absence of Rf1A, Rf1B decreases *orf79* mRNA levels dramatically without generating additional, smaller transcripts (Wang et al., 2006). It was proposed therefore that Rf1B acts in restoration of fertility via a different mechanism than Rf1A by inducing the destabilization of *orf79* dicistronic mRNA (Wang et al., 2006). When both restorers are present, the *atp6/orf79* dicistronic mRNA is preferentially targeted by *Rf1A* (Kazama and Toriyama, 2003; Komori et al., 2004). The inability of Rf1B to destabilize the RNA fragments cleaved by Rf1A suggests that this cleavage also eliminates a recognition sequence in the intercistronic region necessary for Rf1B-dependent RNA degradation (Wang et al., 2006).

Although the exact identity or mechanisms remain unexplored, a number of PPR proteins in other plant CMS systems are thought to act as fertility restorers. In rice, Tang et al. (2014) reported the characterization of Rf4, another PPR protein acting as a restorer by reducing the levels of the wild-abortive CMS conferring transcript WA352 (**Table 1**). Barr and Fishman (2010) have mapped a restorer locus in *Mimulus guttatus* that contains a large cluster of 17 PPR protein genes, suggesting that one of these genes could function in fertility restoration. In sorghum CMS systems, the restorer of fertility locus *Rf1* and *Rf2* also contains a *PPR* gene, which presents high homology with the rice *Rf1* (Jordan et al., 2010). *Rf5*, the locus responsible for restoration of A1 and A2 CMS in sorghum, also contains a cluster of *PPR* genes presenting high homology to *Rf1* in rice (Jordan et al., 2011). Likewise, the *Rf98* locus, restoring the RT98-type CMS in rice, was found to contain a cluster of seven PPR genes. Among these, the *PPR762* gene was found to carry very partial restoration activity and other genes near the *Rf98* locus appeared to be necessary for the full recovery of seed setting (Igarashi et al., 2016).

The involvement of more than one gene in fertility restoration has been previously demonstrated. In the Honglian rice CMS lines, the restoration of fertility also requires a PPR protein. *Rf5*, the restorer of fertility in HL-CMS identified by map based cloning, is identical to *Rf1A* in BT-rice CMS but surprisingly does not restore fertility using the same mechanisms (**Table 1**, Hu et al., 2012). Indeed, Rf5 was not found to be able to bind directly to the CMS conferring transcript, *atp6-orfH79* (Hu et al., 2012) but rather to work in a complex with a glycine-rich protein, a mechanism we will explore later on in this review.

## PPR-RFS Evolve From A *PPR* Gene Subgroup Showing Diversifying Selection

As mentioned earlier, characterization of the restorer locus in BT-rice CMS revealed that it contains nine *PPR* genes (Akagi et al., 2004). The overall level of homology between these nine *PPR* genes suggests a pattern of evolution through local sequence duplication (Akagi et al., 2004). Similar organizations with clusters of *PPR* genes have been observed in the restorer locus of petunia (Bentolila et al., 2002) and radish (Brown et al., 2003; Desloire et al., 2003; Koizuka et al., 2003). In these loci, the restorer of fertility clusters with other restorer of fertility-like *PPR* genes usually presenting a high level of sequence homology with each other (Bentolila et al., 2002; Brown et al., 2003; Akagi et al., 2004). It was suggested that this pattern of clustering in various plants might show diversifying selection acting on *PPR* genes from these regions (Geddy and Brown, 2007; O’Toole et al., 2008).

A diversifying selective pressure as an evolutionary process selects for, rather than against, mutations that would lead to amino acid replacements in the encoded proteins. As a result, plants would adapt to newly emerging sterility inducing genes by developing new *PPR* genes, a process analogous to the gene for gene evolution of disease resistance genes in response to newly emerging pathogen races (Dangl and Jones, 2001). A genome wide distribution analysis of *PPR* genes indicates that although the vast majority of *PPR* genes are dispersed throughout the *A. thaliana* genome (Lurin et al., 2004), a loose cluster of *PPR* genes is present on the long arm of chromosome 1 with 19 genes in close vicinity of each other (Desloire et al., 2003). Subsequently, Geddy and Brown (2007) showed that some *PPR* genes are rarely maintained in the same position or orientation between closely related species (*Brassica napus* vs. *A. thaliana*). Thus some *PPR* genes are “nomadic” in nature, i.e., they migrate from one genomic position to another, and under pressure to alter their sequences, thus creating changes that will diversify the *PPR* gene population. This differs from most other PPR gene families (non-Rf) which have a tendency to select against mutation and thus to conserve the sequence of functional proteins (O’Toole et al., 2008). Additionally, many *PPR* genes lack introns, suggesting their duplication may involve a retrotransposition type process.

Not only are some members of the PPR protein family under diversifying selective pressure but also within a single PPR protein, different amino acids are subject to different degrees of selective pressure. In an extensive study of the PPR protein family in 11 angiosperm species, Fujii et al. (2011) revealed that Rf proteins fall within a specific clade of PPRs. They showed that some members of this clade were subject to diversifying selection, as indicated by synonymous vs. non-synonymous substitution rates, and that, in these cases, the probability of diversifying selection was 5 to 15 times higher at residues 1, 3, and 6 of each PPR motif than at other amino acids of the domain. These amino acid residues were subsequently found to be implicated in the recognition of their target RNA (Barkan et al., 2012; Takenaka et al., 2013; Yagi et al., 2013; Yin et al., 2013). The changes in sequences promoted by this selective pressure are thus predicted to directly target the amino acids responsible for the CMS conferring transcript recognition. This generalized pattern of diversifying selection suggests that this subgroup of PPR proteins are driven to rapidly evolve as sequence-specific RNA binding proteins to accommodate the appearance of new, CMS conferring mitochondrial genes. Thus diversifying selection aids in the creation of novel restorer of fertility genes to silence the newly arisen CMS conferring transcripts.

Fujii et al. (2011) designated the subgroup of PPR proteins encompassing fertility restorers as the Restorer of Fertility-Like (RFL) proteins within the P subfamily of PPR proteins. This analysis showed that most of the non-RFL *PPR* genes from different species form orthologous phylogenetic clusters, suggesting that these proteins are descended from ancestors present in the genome before the species diverged (O’Toole et al., 2008; Fujii et al., 2011). In contrast, *RFL* genes form species-specific paralogous clusters, indicating that these genes have extensively evolved since these species diverged. Despite the rapid evolution of *RFL* genes, monocot and dicot *RFL* proteins still form distinct lines of descent within a single clade of PPRs. Identified PPR restorer proteins from petunia, radish and rice all cluster with the corresponding line of descent within the RFL clade. The overall analysis provides strong support for the hypothesis that *RFL* and *PPR-Rf* genes have a monophyletic origin that precedes the modern monocot-dicot division, and have evolved quickly to produce a separate sub-group of proteins within P-class PPR proteins. Moreover, a significantly greater proportion of *RFL* genes show diversifying selection, as measured by non-synonymous vs. synonymous substitution rates, than what is observed in non-RFL *PPR* genes. Thus, about 10% of *RFL* genes show high probabilities of diversifying selection compared with most other genes in the genome, and in particular, compared with other *PPR* genes (Fujii et al., 2011). These findings suggest the RFL subgroup of *PPR* genes serves as a pool from which new *Rf* genes can emerge.

Characterization of several *Arabidopsis RFL* genes indicated that they represent poorly conserved *PPR* genes even at the species level and that some of them direct non-essential endoribonuclease processing events within conserved mitochondrial transcripts. One of them, *RFL9*, may have evolved in response to a CMS in *Arabidopsis* although the corresponding T-DNA mutants were not found to be male sterile (Arnal et al., 2014). RFL9 clusters with a subgroup of 12 highly similar *Arabidopsis* RFL proteins for which no obvious orthologs can be found even in *A. lyrata* (Fujii and Small, 2011). This indicates that within one species a subgroup of fast-evolving *RFL* genes can emerge in a relatively short evolutionary time period, confirming the predictions of Fujii et al. (2011) at the species level. Within the same 12 RFL proteins subgroup, RFL9 is extremely similar to four other RFL proteins (Arnal et al., 2014). The sequence similarity between these five genes extends from the promoter region through the coding sequence into downstream sequences. The findings suggest active sequence exchanges may be occurring within genes. This, in turn, could promote sequence re-shuﬄing allowing the multiplication and diversification of *RFL* genes in plants in order to create new *RFL* copies (Arnal et al., 2014).

## Non-PPR Fertility Restorer Genes and their Relationships with RF-PPRS

As mentioned previously, restoration of fertility includes several other well-documented mechanisms, which do not involve PPR proteins, at least directly. The processing factors include glycine-rich proteins (GRPs), aldehyde dehydrogenase, acyl-carrier proteins and a peptidase. Beside GRPs, most of these restoration mechanisms act at the metabolic level rather than on the transcripts of the chimerical CMS-conferring genes.

### Glycine-Rich Proteins (GRPs)

Recent studies have opened a new perspective in the fertility restoration field with the characterization of GRP involved in the restoration process of several CMS systems. Hu et al. (2012) first revealed the involvement of GRP162 in the restoration process of CMS-Honglian (HL) in rice (**Table 1**). HL-CMS is associated with the dicistronic transcript *atp6-orfH79* and male fertility can be independently restored by either of two restorer genes, *Rf5* or *Rf6* (Liu et al., 2004). The cloning of *Rf5* revealed that it was identical to *Rf1A* in BT-CMS rice (as discussed previously in this review). However, the fertility restoration function of *Rf5* requires the co-action of a glycine-rich protein, GRP162 (Hu et al., 2012). The presence of GRP162 alone is responsible for a translation inhibition of *orfH79* and allows fertility restoration. GRP162 has two RNA recognition motifs that bind to *orfH79* (Hu et al., 2013) but does not contain any mitochondrial targeting sequence. It has been proposed that Rf5 would recruit GRP162 to the mitochondria by forming a heterodimer in the cytosol so that those two components of a larger restoration complex (RFC) could bind and process CMS-associated transcripts (Hu et al., 2012). Recently, the assembly of the restoration of fertility complex involving Rf5 and GRP162 was shown to require a DUF1620-containing and WD40-like repeat protein (Qin et al., 2016). The RFC was found to measure around 400–500 kDa, so other components of the complex remain to be identified.

In plants, GRP proteins are characterized by the presence of semi-repetitive glycine-rich motifs. The classification of this large protein family depends on their general structure, the arrangement of the glycine repeats as well as the presence of conserved motifs (Mangeon et al., 2010). Of particular interest, class IV GRPs contain an RNA-recognition motif and are known to bind RNAs. The RNA-binding activity of these proteins has been biochemically demonstrated, suggesting that they may be involved in RNA stabilization, processing or transport. Some class IV GRPs also have an RNA-chaperone activity (Mangeon et al., 2010). GRPs act in numerous processes and the specific function of the glycine-rich domain still remains unclear. One can assume, however, that proteins known to be implicated in RNA recognition and processing as GRPs could be involved in fertility restoration as proposed for HL-CMS in rice with GRP162.

Glycine-rich proteins have also been proposed to function as fertility restorers in the absence of PPR proteins. The GRP protein Rf2, the restorer for Lead-Rice CMS (LD-CMS, Itabashi et al., 2011), has been shown to interact with RIF2, an ubiquitin domain-containing protein (**Table 1**, Fujii et al., 2014). The interaction of the two proteins suggests the presence of a large RFC targeting the degradation of the CMS-causing protein and implying a process of fertility restoration that does not include transcript processing or translation inhibition. Like in the BT-CMS, the *orf79* is also the CMS-causing gene of the LD-CMS (Kazama et al., 2016). It is therefore interesting to observe that two different mechanisms have been created to silence *orf79*, one involving a PPR restorer gene (in BT-CMS) and the other not (in LD-CMS).

### Aldehyde Dehydrogenase

Other non-PPR male fertility restorers have been characterized in different crops, all using a fertility restoration process that does not include post-transcriptional modification. *Rf2* in T-CMS in maize (Cui et al., 1996) encodes a mitochondrial aldehyde dehydrogenase (Liu et al., 2001) and has no effect on the expression of the T-CMS associated mitochondrial gene T-*urf13* (**Table 1**). The current model of action of Rf2 proposes that it would act by oxidizing a number of aldehydes to prevent oxidative stress induced by the CMS conferring polypeptide URF13 (Liu et al., 2001). However, *Rf2* also affects normal anther development in normal maize cytoplasm, which carries a non-sterility inducing cytoplasm.

### Acyl-Carrier Proteins

The restoration factor of (CW)-type CMS in rice, Rf17, has been found to encode a protein of unknown function bearing partial homology with acyl-carrier proteins (Fujii and Toriyama, 2009). The function of this restorer protein is unclear but it has been proposed that it would restore fertility by retrograde regulation, i.e., by altering the nuclear response to mitochondrial function. It was shown that the reduced-expression allele of *Rf17* restored fertility in haploid pollen, whereas a normal-expression allele caused pollen lethality in the CW-type CMS (Fujii and Toriyama, 2009). Although there were no indications of Rf17 functions other than the partial acyl carrier protein synthase-like domain, the authors speculated that some metabolic alteration in mitochondria restores pollen fertility, similar to the mechanism in the maize Rf2 system described above (Fujii and Toriyama, 2009).

### Peptidases

In sugar beet (*Beta vulgaris* L.), restoration of fertility of the Owen CMS also does not include a PPR protein. The *Rf* gene of this system was mapped to a region that does not contain any *PPR* genes (Matsuhira et al., 2012). The restoration activity is carried by the *bvORF20* gene, which encodes a mitochondrial-targeted protein exhibiting strong homology with the OMA1-like metallopeptidase (**Table 1**, Kitazaki et al., 2015). It was shown that bvORF20 interacts with the Owen CMS conferring mitochondrial polypeptide, preSATP6 and that *bvORF20* expression correlates with a decrease of a 250 kDa membrane-bound complex containing the preSATP6 protein. It has been proposed that bvORF20 would restore fertility post-translationally by limiting the homo-oligomerization of preSATP6 in mitochondrial membranes.

Furthermore, the CMS-sprite in the common bean has been associated with the mitochondrial gene *pvs-orf239* whose translational product is only found in the reproductive tissues (Abad et al., 1995). A LON-like protease activity associated with the mitochondrial inner membrane was found to be responsible for post-translational proteolytic degradation of ORF239 in vegetative tissue specifically (Sarria et al., 1998). Therefore, a LON-like mitochondrial protease acts as a tissue-specific suppressor of *pvs-orf 239* expression. However, this protease is likely unrelated to the CMS-sprite restorers, which were shown to act by either reducing the amount of the *pvs-orf239* encoding DNA or by affecting the transcript profile of the *pvs* region (Chase, 1994; He et al., 1995).

## *RFL* PPRS and Nuclease Tailoring of Plant Mitochondrial Transcriptome

Perhaps the most interesting aspect of the *RFL* PPR genes of *A. thaliana* is that knockout mutations in most of them have no overt phenotypic consequences and none of these knockout experiments has been found to result in CMS. Only one form of CMS, attributed to a novel mitochondrial gene, *orf117Sha*, has been described for *A. thaliana* (Gobron et al., 2013) and this is an unusual CMS in that, for the two nuclear loci that interact with the causative cytoplasm, maintainer alleles are dominant over restorers. Why then, has this relatively large subfamily of genes been maintained over the generations that have taken place since the origin of the species? A similar question can be asked of the many *RFL* genes in other species that have no clear relationship to nuclear fertility restoration.

Transcription in plant mitochondria is a highly relaxed process (Hammani and Giegeè, 2014), and the production of mature mtRNA species is largely due to post-transcriptional events including splicing, editing and, in particular, nuclease cleavage events. Plant mitochondrial processing exonucleases act predominantly, if not exclusively, in the 3′–5′ direction. A key factor in targeting transcripts for degradation in mitochondria, as in chloroplasts and bacteria is polyadenylation at their 3′ terminus (Lange et al., 2009); such polyadenylated RNAs are then rapidly degraded by mitochondrial 3′ to 5 exonucleases, particularly polynucleotide phosphorylase (Holec et al., 2006).

A common feature of nuclear restorer gene action is the capacity to reduce the abundance of transcripts spanning cognate CMS-associated ORFs concomitant with appearance of transcripts with 5′ termini mapping within the ORF incapable of specifying the CMS-associated polypeptide (Kennell and Pring, 1989; Singh and Brown, 1991; Dill et al., 1997; Menassa et al., 1999; Wise et al., 1999). A likely explanation for this phenomenon is that the restorer conditions endonucleolytic cleavage within the ORF, with the resultant destabilization of the upstream RNA fragment due to the absence of a factor at its 3′ end that protects it from 3′ to 5′ exonuclease activity (Brown, 1999). Such a process resembles the mechanism by which several *Arabidopsis* Rf-like PPR proteins act to generate 5′ termini in the 5′UTR of standard mitochondrial genes (Jonietz et al., 2010; Hölzle et al., 2011).

Given the similarity between the mode of action of some nuclear restorer genes and Rf-like PPRs, it is conceivable that some, perhaps many, of the Rf-like PPRs may participate in the degradation processes that help define the stable mitochondrial transcriptome. According to this view, these proteins would mediate cleavage in, and thereby destabilize RNAs derived from the large portion of the mitochondrial genome that is non-functional but transcriptionally active. The Rf-like PPR *RFL9* is particularly interesting in this respect since it conditions cleavage within the non-functional mitochondrial ORF *orf240a*, as well as within the functional *rps3* gene (Arnal et al., 2014). This reduces the levels of functional *rps3* transcripts, suggesting that the transcript destabilization process can tolerate a certain degree of flexibility with respect to the transcripts of functional gene. Similarly, the *B. napus Rfn* gene mediates cleavage events that destabilize the CMS-associated *orf222* gene as well as two functional mitochondrial genes, *nad4* and *ccmF_N2_* (Singh et al., 1996). Although expression of a full length *orf240a* transcript as observed in *rfl9* mutants, does not result in male sterility, this ORF does share significant sequence similarity to the ORFs associated with CMS in *B. napus, orf222* and *orf224*, and it has been implicated in specifying an alloplasmic CMS in *Brassica*/*Arabidopsis* cybrids in which *Arabidopsis* is the cytoplasmic donor. These observations suggest that an *orf240a*-like sequence served as an ancestor to modern *Brassica* CMS-associated genes and that variety of factors may influence the capacity of such sequences to cause male sterility.

Holec et al. (2006) reported that several polyadenlylated RNAs that accumulated in *Arabidopsis* plants down regulated for PNPase corresponded to non-functional ORFs. The authors suggest that one function of the transcript degradation process may be to prevent the translation of non-functional ORFs, which could have a negative effect on mitochondrial function. In support of this view, it has recently been reported that the *Arabidopsis* RFL2 protein cleaves within the transcript of one such ORF, *orf291*, thereby destabilizing the ORF transcript (Fujii et al., 2016). This cleavage requires the participation of the tRNA processing endonuclease RNase P, and the authors suggest that RFL2 binding may induce the formation of a tRNA-like structure in the *orf291* transcript that serves as a substrate for RNase P. It is conceivable that other *RFL* genes in *Arabidopsis* may play a role in defining the mitochondrial transcriptome by mediating transcript cleavage and destabilizing events in non-functional transcribed regions; these could even include some *RFL* genes that play a role in mediating 5′ transcript end formation. Such genes would trigger the removal of transcripts having a potentially negative function on mitochondrial function such as anti-sense RNAs and non-functional ORFs. It is expected that such proteins would change their targets as changes occurred in the mitochondrial genome, consistent with the diversifying selection pattern observed for *RFL* genes. Thus, while the primary function of this group of proteins may be involved with the mitochondrial transcript degradation processes, they could also serve as a pool of rapidly evolving proteins from which new Rf factors could emerge.

## Conclusion

This review explores the functional specificities of the different restorers of fertility identified in plants so far. It highlights the wide array of mechanisms guiding fertility restoration, which are in most cases unique to each CMS. PPR proteins represent the most frequent protein class among identified Rfs. PPR proteins exhibit ideal characteristics to evolve into restorer of fertility when the mechanism of restoration implies a post-transcriptional action on mitochondrial gene expression. They have the ability to bind specific RNAs with high specificity and to impact the processing, the stability or the expression of their target RNA in several ways. To suppress male sterility, Rf PPRs most often induce endoribonucleolytic cleavage or act as a physical barrier to block translation of CMS conferring transcripts. Several studies now support clearly that certain Rf PPRs interact with other types of protein partners, like glycine-rich proteins, to achieve their function. The diversifying evolutionary pressures acting on *PPR* genes in general and notably on the Rf-like PPR sub-group greatly accelerate the emergence of novel *PPR* alleles in plant populations which can be selected as fertility restorers when they bind to and impact the expression of CMS-causing transcripts. The role of several *RFL* PPRs is consistent with the premise that these proteins may play a more general role in destabilizing non-functional transcripts. The versatility by which PPR proteins can block the expression of CMS transcripts and the rapidity of adaptation of *Rf-like* genes to newly arising RNA sequences likely explain why fertility restorer genes often correspond to *PPR* genes. Recent data indicate that glycine-rich proteins may act in concert with PPR proteins to suppress CMS transcript expression. Some of these GRPs have RNA binding activity and thereby may assist PPR proteins to bind CMS transcripts in a productive way. The mode of action of other GRPs in fertility restoration needs to be clarified but they may stabilize PPR/RNA complex and thus accelerate the emergence of new PPR-Rfs.

## Author Contributions

The text of this review was mostly written by LG with the help of HM. GB wrote the evolutionary general statements presented at the end of the review and improved the English of the manuscript.

## Conflict of Interest Statement

The authors declare that the research was conducted in the absence of any commercial or financial relationships that could be construed as a potential conflict of interest.
